# High Rates of Uncontrolled Blood Pressure in Malawian Adults Living with HIV and Hypertension

**DOI:** 10.5334/gh.1081

**Published:** 2021-12-06

**Authors:** Risa M. Hoffman, Florence Chibwana, Daniel Kahn, Ben Allan Banda, Linna Phiri, Mayamiko Chimombo, Chiulemu Kussen, Hitler Sigauke, Agnes Moses, Joep J. van Oosterhout, Sam Phiri, Jesse W. Currier, Judith S. Currier, Corrina Moucheraud

**Affiliations:** 1 Department of Medicine and Division of Infectious Diseases, David Geffen School of Medicine at the University of California, Los Angeles, California, US; 2 Department of Health Policy and Management, Fielding School of Public Health at the University of California, Los Angeles, California, US; 3 Partners in Hope, Lilongwe, MW; 4 Department of Medicine, David Geffen School of Medicine at the University of California, Los Angeles, California, US; 5 Department of Medicine, Division of Cardiology, David Geffen School of Medicine at the University of California, Los Angeles, California, US

**Keywords:** hypertension, hypertension control, HIV, antiretroviral therapy, adherence, non-communicable diseases

## Abstract

**Background:**

Hypertension is among the most commonly diagnosed non-communicable diseases in Africa, and studies have demonstrated a high prevalence of hypertension among individuals with HIV. Despite high prevalence, there has been limited attention on the clinical outcomes of hypertension treatment in this population.

**Objective:**

We sought to characterize rates of and factors associated with blood pressure control over one year among individuals on antiretroviral therapy (ART) and antihypertensive medications.

**Methods:**

We performed a prospective observational cohort study at an HIV clinic in Malawi. We defined uncontrolled hypertension as a systolic blood pressure ≥140 mm Hg and/or diastolic blood pressure ≥90 mm Hg at two or more follow-up visits during the year, while controlled hypertension was defined as <140 mm Hg systolic and <90 mm Hg diastolic at all visits, or at all but one visit. We calculated an antihypertensive non-adherence score based on self-report of missed doses at each visit (higher score = worse adherence) and used rank sum and chi-square tests to compare sociodemographic and clinical factors (including adherence) associated with blood pressure control over the year.

**Results:**

At study entry, 158 participants (23.5%) were on antihypertensive medication; participants had a median age of 51.0 years, were 66.5% female, and had a median of 6.9 years on ART. 19.0% (n = 30) achieved blood pressure control over the year of follow-up. Self-reported non-adherence to hypertension medications was the only factor significantly associated with uncontrolled blood pressure. The average non-adherence score for those with controlled blood pressure was 0.22, and for those with uncontrolled blood pressure was 0.61 (p = 0.009).

**Conclusions:**

Adults living with HIV and hypertension in our cohort had low rates of blood pressure control over one year associated with self-reported non-adherence to antihypertensive medications. Given the high prevalence and incidence of hypertension, interventions to improve blood pressure control are needed to prevent associated long-term cardio- and cerebrovascular morbidity and mortality.

## Introduction

Non-communicable diseases (NCDs) are the leading cause of death worldwide and have become increasingly prevalent in low and middle-income countries (LMICs) [1]. Over 80% of premature NCD-related deaths now occur in LMICs [1,2]. Cardiovascular risk factors are rising in LMICs due to unhealthy lifestyle changes related to urbanization and globalization, including physical inactivity; diets high in refined grains, added sugar, and unhealthy fats; and a related epidemic of obesity [3]. In sub-Saharan Africa, NCDs are predicted to surpass the combined mortality from maternal, neonatal, and nutritional diseases by 2030 [4].

Countries in sub-Saharan Africa with a high burden of NCDs also have the highest prevalence of HIV globally, which is notable because HIV infection and antiretroviral therapy (ART) are associated with increased risk of developing hypertension, dyslipidemia, diabetes, and cardiovascular disease through a combination of chronic inflammation and immune activation associated with HIV itself, toxicities of treatment, and unhealthy lifestyle changes [5]. While the majority of published data about HIV and NCDs is from high-income countries [6], there is a growing body of literature that suggests NCDs are also highly prevalent in people living with HIV in sub-Saharan Africa [7,8,9]. Addressing the HIV-NCD syndemic will be critical to maintaining the gains in life expectancy provided through lifelong ART [10].

Hypertension is among the most commonly diagnosed NCDs in sub-Saharan Africa, and studies have demonstrated a high prevalence of hypertension among individuals with HIV (11%–46%) [11,12,13]. Measuring and treating blood pressure is a low-cost, feasible intervention in resource-limited settings; therefore, hypertension screening and treatment has been implemented within many HIV treatment programs in Africa. Despite extensive literature on improved HIV outcomes related to ART, there has been limited attention on the clinical outcomes of hypertension treatment in this population. A small number of studies from sub-Saharan Africa have followed individuals with HIV and hypertension prospectively, and demonstrated low rates of blood pressure control, including 46% controlled in Uganda over three years of follow-up [14] and 30% controlled among individuals in Malawi after six months of follow-up [15]. To date, only one prospective study has evaluated factors associated with uncontrolled blood pressure among people living with HIV in Africa, and identified male sex and age >50 years as risk factors [14]. The role of adherence to antihypertensive medication in people living with HIV who also take ART has not been evaluated in prospective studies of blood pressure control.

Malawi is a small country in Africa with a robust national HIV treatment program, which incorporated screening and treatment for hypertension into its national HIV guidelines in 2016 [16]. We performed a longitudinal cohort study of adult HIV patients on ART at an urban health clinic in Malawi. Our primary study objective was to characterize blood pressure control over one year among individuals on antihypertensive medication at baseline. Secondary objectives included evaluating factors associated with hypertension control, including adherence, and assessing the incidence of hypertension among those not known to be hypertensive at baseline.

## Methods

We performed a prospective observational cohort study at Partners in Hope Medical Center, a faith-based non-governmental organization located in Lilongwe, Malawi. The clinic provides free HIV care in the urban setting of Lilongwe, Malawi to an active cohort of over 5,000 patients on ART. During the time of the study, hypertension screening was provided free of charge, and medications for hypertension could be purchased at Partners in Hope (or other private health facilities or pharmacies) or acquired for free at government-operated health facilities. Hypertension management follows a specific algorithm outlined by the Malawi Standard Treatment Guidelines: first line therapy with a thiazide diuretic (typically hydrochlorothiazide) and sequential additions of medications if blood pressure control is not achieved (calcium channel blocker, followed by an angiotensin converting enzyme inhibitor, followed by a beta-blocker) [17]. Lifestyle recommendations are recommended for mild hypertension, with initiation of therapy if these are unsuccessful. On average, at the time under study, individuals on ART attended clinic every three months for clinical evaluations and ART refills, had routine viral load testing every two years, and received blood pressure measurement and management at each ART visit by providers working in the HIV clinic. At Partners in Hope, the majority of routine clinical care in the HIV clinic is provided by clinical officers (who complete three years of technical training followed by a one-year internship).

### Recruitment, screening, and enrollment

Individuals were approached while waiting to receive routine HIV care and asked if they would be interested in participating in a study about hypertension. Those who expressed interest were taken to a private room to hear more information about the study, provide oral consent for screening, and if eligible, provided written informed consent to participate in the study. Individuals were eligible if they were ≥18 years of age, had been receiving ART from Partners in Hope for at least one year at the time of study entry, planned to remain a patient at Partners in Hope for at least the next one year, and did not have any clinical condition that required urgent medical attention on the day of study entry (such that they were deemed stable to have a study visit on that day).

### Study procedures

At the entry visit, participants completed a survey about HIV clinical history and adherence to ART, lifestyle (diet, exercise, substance use), and, if on medications for hypertension, antihypertensive medication adherence, side effects, and barriers to taking antihypertensive medications (with survey questions about barriers based on questions previously validated at Partners in Hope for patients on ART). Participant charts were reviewed at entry to confirm current hypertension medications and to document HIV disease characteristics (HIV diagnosis, duration on ART, ART regimen, and viral load within 12 months prior to study entry, if available).

All participants had blood pressure measured and recorded at least twice during the course of the entry visit: one time at the patient registration area (part of routine care at the HIV clinic) and one time by the study nurse (at the beginning of the study visit). For those with an elevated blood pressure (based on the Malawi definition of hypertension: ≥140 mm Hg systolic and/or ≥90 mm/Hg diastolic) on either of the first two measurements, a third blood pressure was obtained at the end of the study visit. All measured blood pressures were recorded in the study record. Blood pressure equipment was calibrated weekly and refresher trainings on proper measurement of blood pressure were provided to staff at the beginning of the study and then quarterly during study follow-up.

All participants were followed for one year. At every routine ART visit at Partners in Hope (approximately every three months as per standard of care at the time under study), the participant had blood pressure measured as described above, and completed a brief survey about adherence to ART, lifestyle (diet, exercise, substance use), and if on antihypertensive medication, adherence, side effects, and barriers to taking these medications. The participant chart was reviewed for antihypertensive medications.

The target sample size for the overall cohort was 700 with the assumption that approximately 30% would have hypertension based on regional data [18], and that this sample size would allow for a large enough population to explore factors associated with uncontrolled hypertension based on rates of blood pressure control from other countries in the region [14,15].

The study was reviewed and approved by the Malawi National Health Sciences Research Committee and the University of California, Los Angeles Institutional Review Board.

### Variable definitions

Given a lack of consistent medical record documentation related to a diagnosis of hypertension and challenges with the ability of individuals to self-report a previous diagnosis of hypertension, participants were defined as hypertensive based on records of active prescriptions for antihypertensive medication at the entry visit. For classification of severity of hypertension, we used the 2015 Malawi National Guidelines, as follows: mild hypertension as a systolic blood pressure 140–159 mm Hg and/or diastolic blood pressure 90–99 mm Hg; moderate hypertension as a systolic blood pressure 160–179 mm Hg and/or diastolic blood pressure 100–109 mm Hg; and severe hypertension as a systolic blood pressure ≥180 mm Hg and/or diastolic ≥110 mm Hg [17]. For all analyses, we used the average of all blood pressures taken at each visit. We defined uncontrolled hypertension as a systolic blood pressure ≥140 mm Hg and/or diastolic blood pressure ≥90 mm Hg at two or more follow-up visits during the year, while controlled hypertension was defined as <140 mm Hg systolic and <90 mm Hg diastolic at all visits, or at all but one visit, during the one year of follow-up. Participants were defined as having incident hypertension if they were not on antihypertensive medication at baseline and had ≥2 follow-up visits over the year of follow-up with a blood pressure ≥140 mm Hg systolic and/or ≥90 mm Hg diastolic.

Main individual-level factors explored for associations with blood pressure control and incident hypertension were sociodemographic factors (age, gender, educational attainment), clinical parameters (duration on ART, viral load, body mass index, comorbidity with diabetes based on participant self-report), health behaviors and non-adherence score. The non-adherence score was calculated based on respondents’ self-reported average weekly adherence to antihypertensive medication since their last visit: miss less than once per week, miss once per week, miss two or three times per week, or miss more than three times per week. These responses were assigned between zero and three points, corresponding to these response options. The non-adherence score was each respondent’s mean number of points across all rounds of follow-up visits, with a higher value indicating poorer adherence to antihypertensive medication. For health behaviors, current cigarette use was defined as self-report of smoking, regardless of frequency, and alcohol use was based on self-report of active drinking, regardless of type or frequency. Sedentary lifestyle was defined as spending more than half of the day seated during most days per week in the past month. Daily added salt was determined based on participant response to the question: ‘Do you add salt to your food on a daily basis?’

### Statistical analysis

Summary statistics were generated to describe characteristics of participants at baseline, overall and stratified by sex. We used rank sum and chi-square tests (for continuous and categorical variables, respectively) to compare factors associated with blood pressure control over the one year among all individuals on treatment for hypertension at baseline, and to evaluate factors associated with hypertension incidence among those without hypertension at baseline. Finally, a multivariate analysis was performed to evaluate blood pressure control among all individuals on treatment for hypertension at baseline. For all statistical analyses, significance was defined as p < 0.050.

Since no standard definitions for defining longitudinal hypertension control exist in Malawi, we performed a sensitivity analysis for factors associated with hypertension control using a strict definition, which required blood pressure at all visits <140 mm Hg systolic and <90 mm Hg diastolic versus uncontrolled, defined as any blood pressure during the year ≥140 mm Hg systolic and/or ≥90 mm Hg diastolic.

## Results

We enrolled 704 adults on ART in the study between November 2015 and October 2017. Thirty-three individuals (4.7%) were excluded from analyses because they were lost from the cohort before two follow-up visits were completed (23 lost to follow-up, 7 transferred to other clinics, 3 died) leaving a total of 671 individuals in the observational cohort study.

Table 1 describes sociodemographic and clinical characteristics of the cohort at baseline overall and by sex. At entry, the median age of participants was 44 years (IQR 39, 52) and 51.7% (n = 347) were female. The median duration on ART at entry was 5.9 years (IQR 4.1, 8.0) and the majority of participants (78.5%, n = 527) were on first-line ART with efavirenz/lamivudine/tenofovir, the standard of care regimen at the time under study. ART adherence at entry was high, with 96.1% reporting never missing doses or missing doses less than once a week, 3.0% reported missing one dose per week, and <1% reporting missing more than one dose per week. Of 294 individuals with a viral load reported in the year prior to study entry, 95.6% (n = 281) were suppressed (threshold < 1000 copies/mL).

**Table 1:** Baseline characteristics of the study population.

	Overall n = 671	Male n = 324	Female n = 347	p-value

Median age (IQR)	44 (39, 52)	46 (39, 53)	43 (38, 52)	0.022
Median years on antiretroviral therapy (IQR)	5.9 (4.1, 8.0)	5.5 (3.8, 7.8)	6.3 (4.3, 8.2)	0.007
Antiretroviral therapy regimen, n (%)				
*NNRTI-based*	637 (94.9%)	310 (95.7%)	327 (94.2%)	0.341
*PI-based*	33 (4.9%)	13 (4.0%)	20 (5.8%)	
*Missing*	1 (0.2%)	1 (0.3%)	0 (0.0%)	
Highest level education completed, n (%)				
*Primary school or less*	385 (57.4%)	169 (52.2%)	216 (62.2%)	0.030
*Secondary*	193 (28.8%)	104 (32.1%)	89 (25.7%)	
*Beyond secondary*	93 (13.9%)	51 (15.7%)	42 (12.1%)	
Cigarette smoking^a^, n (%)	23 (3.4%)	23 (7.1%)	0 (0.0%)	<0.001
Alcohol use^b^, n (%)	80 (11.9%)	69 (21.3%)	11 (3.2%)	<0.001
Sedentary lifestyle^c^, n (%)	172 (25.6%)	105 (32.4%)	67 (19.3%)	<0.001
Daily added salt to diet^d^, n (%)	658 (98.1%)	316 (97.5%)	342 (98.6%)	0.334
Median Body Mass Index kg/m^2^ (IQR)	23.2 (20.4, 26.8)	21.7 (19.8, 24.3)	25.2 (22, 28.4)	<0.001
Undetectable viral load copies within 12 months of baseline visit^e^ (<1,000 copies/mL), n (%)	281 (95.6%)	146 (96.7%)	135 (94.4%)	0.341
Diabetes^f^, n (%)	20 (3.0%)	8 (2.5%)	12 (3.5%)	0.456
Blood pressure at baseline, n (%)				
*<140/90 mm Hg*	416 (62.0%)	215 (66.4%)	201 (57.9%)	0.042
≥*140–159 and/or* ≥ *90–99 mm Hg*	144 (21.5%)	66 (20.4%)	78 (22.5%)	
≥*160 and/or* ≥ *100 mm Hg*	111 (16.5%)	43 (13.3%)	68 (19.6%)	
Taking antihypertensive medication at baseline, n (%)				
*No*	513 (76.5%)	271 (83.6%)	242 (69.7%)	<0.001
*Yes*	158 (23.5%)	53 (16.4%)	105 (30.3%)	

NNRTI: non-nucleoside reverse transcriptase inhibitor (n = 527 efavirenz and n = 110 nevirapine). PI: protease-inhibitor (n = 1 lopinavir/ritonavir and n = 32 atazanavir/ritonavir). ^a^ Based on self-report of current tobacco smoking, regardless of duration or number of cigarettes per day. ^b^ Alcohol use defined as any ‘yes’ response to survey question ‘Do you drink alcohol?’, regardless of frequency or quantity. ^c^ Sedentary lifestyle defined as spending more than half of the day seated during typical days in the past month. ^d^ Based on self-report of adding salt to food on a daily basis. ^e^ Out of 294 with a viral load recorded. ^f^ Based on self-report.

Diabetes was present in 3.0% of the population (n = 20) and rates of tobacco and alcohol use were low (3.4% [n = 23] and 11.9% [n = 80], respectively). Women were slightly younger than men (43 versus 46 years, p = 0.022), were less likely to report smoking (0 women versus 7.1% of men, p < 0.001) and alcohol use (3.2% versus 21.3%, p < 0.001), had higher body mass index (25.2 versus 21.7, p < 0.001), and were less likely to report a sedentary lifestyle (19.3% versus 32.4%, p < 0.001). Most participants had three follow-up visits after study entry (74.7%, n = 501). The remainder had either two (15.2%, n = 102) or four visits (10.1%, n = 68) after entry.

**Figure 1:**
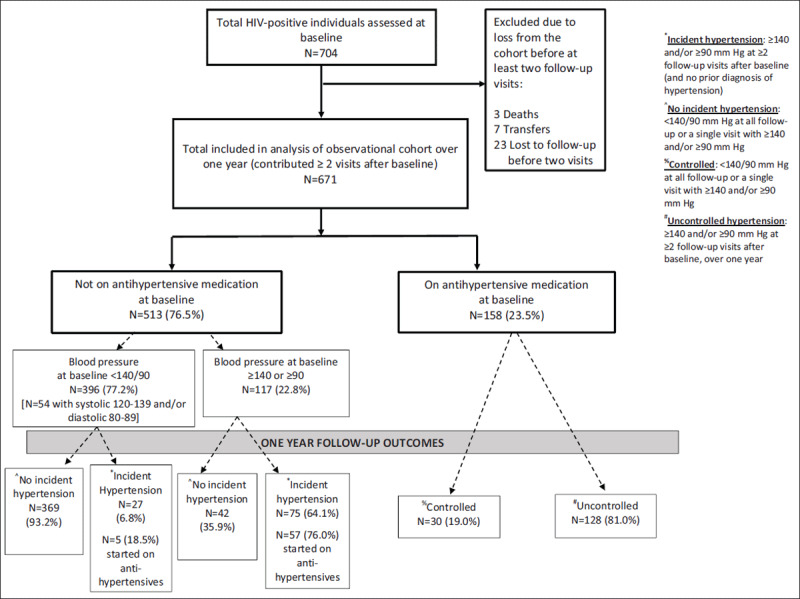
Flow diagram showing participants included in the observational cohort and outcomes from entry to one year of follow-up.

**Figure 2:**
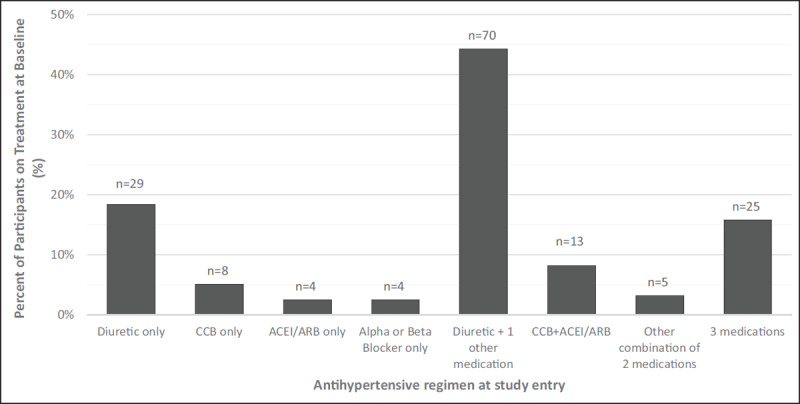
Antihypertensive medications at baseline among participants on treatment (n = 158). Notes: For patients on a diuretic and one other medication, the other medication was a calcium channel blocker (CCB) for 59% (n = 41); alpha or beta blocker for 26% (n = 18), or an angiotensin-converting enzyme inhibitor (ACEI) or angiotensin receptor blocker (ARB) for 16% (n = 11). Among regimens of three medications, 92% (n = 23) included a diuretic and the most common combinations was a diuretic + CCB + ACEI or ARB (72%, n = 18). Medications by class included: Diuretics: hydrochlorothiazide, chlorthalidone Calcium channel blockers: nifedipine, amlodipine, hydralazine ACEI/ARB: enalapril, captopril, telmisartan, losartan Beta blockers and alpha blockers: propranolol, atenolol, methyldopa.

Figure 1 describes the observational cohort and study outcomes for both blood pressure control and incident hypertension. At study entry, 158 participants (23.5%) were known to be hypertensive based on the use of one or more antihypertensive medications, and were included in the group followed longitudinally to assess blood pressure control. Antihypertensive medication regimens at baseline are described in Figure 2. Dual therapy with a diuretic plus a calcium channel blocker was the most common regimen (44.3%, n = 70), followed by monotherapy with a diuretic (18.4%, n = 29). Among those on treatment for hypertension at baseline, approximately 72% of individuals (n = 114) refilled hypertension medications at Partners in Hope. The most common reasons for refilling elsewhere were related to medications being free or less expensive at another location (59.0%) or it was too far to come back to Partners in Hope for refills (29.5%).

**Figure 3:**
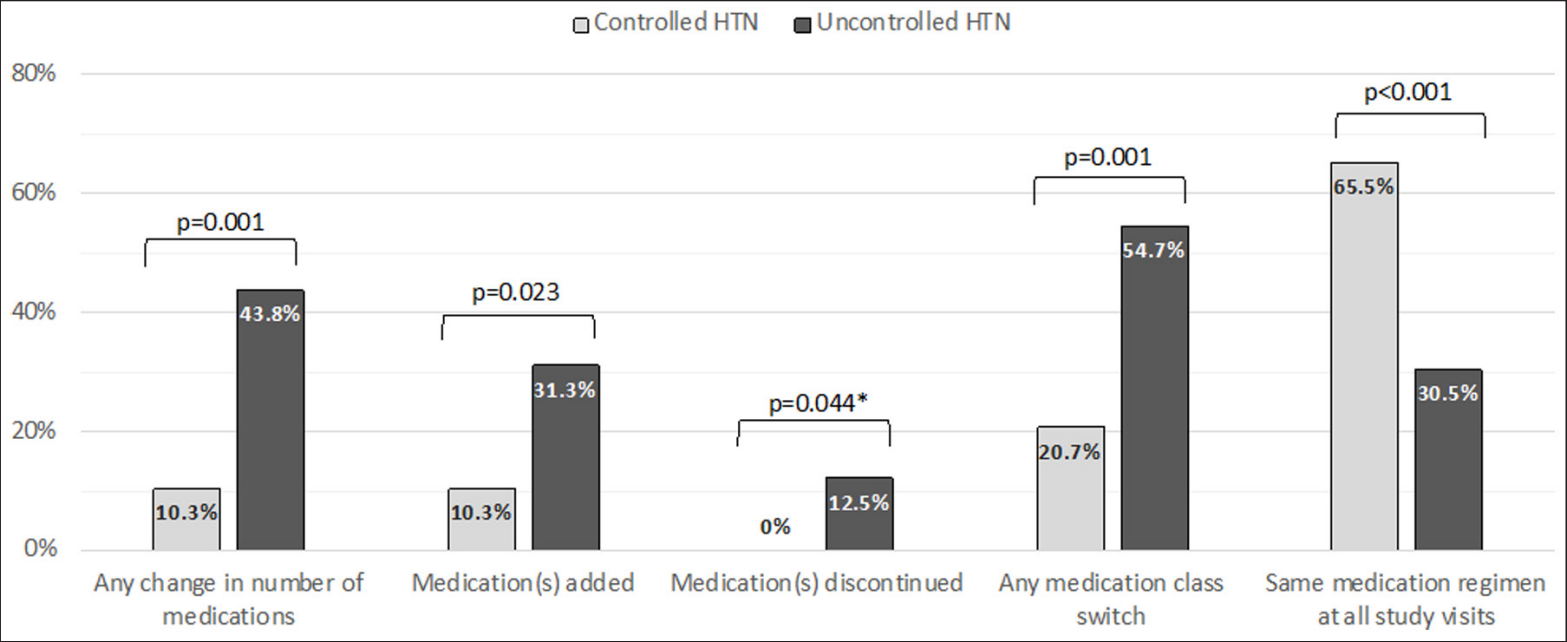
Changes to anthipertensive medications from entry to last visit on study among those on treatment at entry (n = 157). Note: Data missing one participant. P-values indicate Pearson’s chi-square results or Fisher’s exact test.

Of those on antihypertensive medication at entry, 19.0% (n = 30) achieved blood pressure control over the year of follow-up. Those with uncontrolled blood pressure were more likely to have medication class switches (54.7% versus 20.7%), medication additions (31.3% versus 10.3%), and discontinuations (12.5% versus 0%) (Figure 3). Supplementary Figure 1 depicts specific antihypertensive medication regimen changes between entry and the end of the one year of follow-up among those with and without blood pressure control.

Approximately half of the participants on antihypertensive medication at entry had a non-adherence score of zero (82/157, 52.2%). This was more common among those with blood pressure control (21/29, 72.4%) compared to those without control (61/128, 47.7%). Only three individuals had a non-adherence score higher than two and all had uncontrolled blood pressure (Table 2).

**Table 2:** Antihypertensive medication non-adherence scores* over one year of follow-up among individuals with hypertension on at least one medication at baseline, comparing those with controlled and uncontrolled hypertension.

Non-adherence score*	Overall** n = 157	Hypertension	

		Controlled^^^ n = 29	Uncontrolled^&^ n = 128

0	82 (52.2%)	21 (72.4%)	61 (47.7%)
0.1–1.0	46 (29.3%)	6 (20.7%)	40 (31.3%)
1.1–2.0	26 (16.6%)	2 (6.9%)	24 (18.8%)
2.1–3.0	3 (1.9%)	0 (0.0%)	3 (2.3%)

* Higher score indicates higher level of non-adherence. Average weekly adherence to antihypertensive medication since last visit was self-reported at each visit and scored as follows: 0 points for missing medications less than once per week; 1 point for missing medication once per week; 2 points for missing medication two to three times per week; and 3 points for missing medication more than three times per week. Non-adherence score calculated as mean number of points per respondent across all follow-up visits. ** Data from self-reported antihypertensive medication adherence at all visits after baseline; Adherence data missing on 1 participant. ^^^ Controlled: blood pressure (<140 systolic and <90 mm Hg diastolic) at every visit or with only a single visit with elevated blood pressure above this level during one year of follow-up. ^&^ Uncontrolled: ≥2 visits during the one year of follow-up with a blood pressure ≥140 systolic and/or ≥90 mm Hg diastolic.

Approximately 20% (n = 102) of participants not known to be hypertensive at study entry (based on prescription data) developed hypertension over one year of follow-up. Of these, 26.5% (n = 27) had a blood pressure < 140/90 mm Hg at entry, and 73.5% (n = 75) had a systolic blood pressure ≥140 mm Hg and/or diastolic blood pressure ≥90 mm Hg. Of those with incident hypertension, 62 (60.8%) were started on antihypertensive medication. Of those not started on antihypertensive medication, 82.5% had mild hypertension at all visits. Blood pressure control could not be assessed among those started on medications due to limited follow-up time within this group.

### Antihypertensive medication side effects

Side effects from antihypertensive medication were reported by 99 of 220 individuals (45.0%) taking these medications over the course of study follow-up and are described in Table 3. The most commonly reported side effects included headache (27.7%), dizziness (23.6%), and weakness (19.1%). Women were more likely to report any side effect compared to men (50.7% versus 35.4%, p = 0.027) and were more likely to report headache (34.8% versus 15.9%, p = 0.002), dizziness (29.7% versus 13.4%, p = 0.006), and weakness (23.9% versus 11.0%, p = 0.018), while men were more likely to report sexual dysfunction (7.3% for men and 0% for women, p = 0.001). Seven participants (3.2%) said that the side effects were bothersome enough to interfere with taking medications daily.

**Table 3:** Summary of side effects reported by individuals on antihypertensive medication at any timepoint during follow-up (n = 99).

Side Effects	Overall	Female	Male	p-value

Any side effect	99 (45.0%)	70 (50.7%)	29 (35.4%)	0.027
Headache	61 (27.7%)	48 (34.8%)	13 (15.9%)	0.002
Dizziness	52 (23.6%)	41 (29.7%)	11 (13.4%)	0.006
Weakness	42 (19.1%)	33 (23.9%)	9 (11.0%)	0.018
Swelling of legs and/or feet	19 (8.6%)	14 (10.1%)	5 (6.1%)	0.301
Sexual dysfunction	6 (2.7%)	0 (0.0%)	6 (7.3%)	0.001
Dry cough	5 (2.3%)	4 (2.9%)	1 (1.2%)	0.419

### Barriers to taking antihypertensive medication

Of those on antihypertensive medication at baseline, 74/158 (46.8%) reported one or more barriers to adherence. Among the reported barriers at baseline, the most common were not having enough money to purchase antihypertensive medications (58.1%, n = 43), difficulty remembering to take medications daily (39.2%, n = 29), lack of funds for transport to clinic for refills (36.5%, n = 27), and feeling like the medications were no longer needed due to good health (29.7%, n = 22) (Table 4). Barriers to taking antihypertensive medication were re-assessed at each follow-up visit, with individuals asked about the most important barrier at each of these visits. Remembering to take medication every day and not having enough money to purchase medication were consistently ranked as the most important barriers over the course of the one year of follow-up.

**Table 4:** Barriers to adherence to antihypertensive medication reported at baseline among adults with HIV and hypertension in Lilongwe, Malawi (n = 74 reporting any barrier).

Barrier*	n (%)

You do not have enough money to buy the high blood pressure medication.	43 (58.1%)
Remembering to take the medication everyday.	29 (39.2%)
You do not have enough money for transport to and from clinic.	27 (36.5%)
You do not feel sick and do not think you need medications.	22 (29.7%)
It is difficult to take medicine every day because of other duties, such as taking care of children/family household.	7 (9.5%)
You do not have enough time to go to the clinic for follow-up visits and refills.	6 (8.1%)
You wanted to see a traditional healer, pastor, spiritual leader and/or take traditional treatments instead.	1 (1.4%)

* Participants could report more than one barrier.

### Factors associated with uncontrolled blood pressure among participants on antihypertensive medication at baseline

Self-reported non-adherence to antihypertensive medications was significantly associated with uncontrolled blood pressure among those who were classified as hypertensive at baseline (i.e., taking medication): the average adherence score was 0.22 for those with controlled blood pressure and 0.61 for those with uncontrolled blood pressure (p = 0.009) (Table 5). Although individuals with uncontrolled hypertension were more likely to report alcohol use (7.0%, versus 0%) and a sedentary lifestyle (21.9%, versus 16.7%), these were not statistically significant. There were no apparent associations between hypertension control and sex, duration on ART, diabetes, or body mass index. Refilling antihypertensive medications at Partners in Hope versus another location was not associated with blood pressure control. In a multivariate analysis controlling for age, gender, average non-adherence score, body mass index, diabetes, and lifestyle, only the average non-adherence score was significantly associated with uncontrolled blood pressure among those with hypertension at entry (adjusted odds ratio 2.69, 95% confidence interval 1.11–6.53, p = 0.028).

**Table 5:** Factors associated with uncontrolled blood pressure over one year of follow-up among individuals with hypertension on at least one antihypertensive medication at baseline.

	Overall n = 158	*Controlled n = 30	**Uncontrolled n = 128	p-value

Median age (IQR)	51.0 (44, 57)	51.5 (44, 55)	51.0 (44, 57.5)	0.838
Female sex, n (%)	105 (66.5%)	19 (63.3%)	86 (67.2%)	0.687
Median years on antiretroviral therapy (IQR)	6.9 (4.8, 9.0)	8.7 (5.4, 9.8)	6.8 (4.5, 8.9)	0.094
Highest level education completed, n (%)				
*Primary school or less*	84 (53.2%)	18 (60.0%)	66 (51.6%)	0.645
*Secondary*	45 (28.5%)	8 (26.7%)	37 (28.9%)	
*Beyond secondary*	29 (18.4%)	4 (13.3%)	25 (19.5%)	
Cigarette smoking^a^, n (%)	0 (0.0%)	0 (0.0%)	0 (0.0%)	n/a
Alcohol use^b^, n (%)	9 (5.7%)	0 (0.0%)	9 (7.0%)	0.135
Sedentary lifestyle^c^, n (%)	33 (20.9%)	5 (16.7%)	28 (21.9%)	0.528
Daily added salt to diet^d^, n (%)	151 (95.6%)	29 (96.7%)	122 (95.3%)	0.746
Average antihypertensive non-adherence score^e^	0.54	0.22	0.61	0.009
Mean Body Mass Index kg/m^2^ (IQR)	25.5 (21.9, 29.4)	27.1 (22.9, 30.6)	25.4 (21.1, 29.4)	0.334
Undetectable viral load copies within 12 months of baseline visit (<1,000 copies/mL), n (%)^f^	64 (97.0%)	11 (100%)	53 (96.4%)	0.521
Diabetes^g^, n (%)	10 (6.3%)	2 (6.7%)	8 (6.3%)	0.906
Refilled at PIH^h^, n (%)	114 (72.2%)	23 (76.7%)	91 (71.1%)	0.540

* Controlled: blood pressure (<140 systolic and <90 mm Hg diastolic) at every visit or with only a single visit with elevated blood pressure above this level during one year of follow-up. ** Uncontrolled: ≥2 visits during the one year of follow-up with a blood pressure ≥140 systolic and/or ≥90 mm Hg diastolic. ^a^ Based on self-report of current tobacco smoking, regardless of duration or number of cigarettes per day. ^b^ Alcohol use defined as any ‘yes’ response to survey question ‘Do you drink alcohol?,’ regardless of frequency or quantity. ^c^ Sedentary lifestyle defined as spending more than half of the day seated during typical days in the past month. ^d^ Based on self-report of adding salt to food on a daily basis. ^e^ Higher score indicates a higher level of non-adherence over the one year of follow-up; score can range from 0–3. Average weekly adherence to antihypertensive medication since last visit was self-reported at each visit and scored as follows: 0 points for missing medications less than once per week; 1 point for missing medication once per week; 2 points for missing medication two to three times per week; and 3 points for missing medication more than three times per week. Non-adherence score calculated as mean number of points per respondent across all follow-up visits. ^f^ Among 66 people with recent viral load available. ^g^ Self-reported. ^h^ Refilled ≥1 time at PIH during follow-up (compared to never at PIH).

In a sensitivity analysis in which blood pressure control was defined as <140/90 mm Hg at all visits and uncontrolled as any visit with a blood pressure ≥140 mm Hg and/or ≥90 mm Hg, no statistically significant associations were identified; however, there was a trend towards higher non-adherence scores among those in the uncontrolled group (0.57 in those uncontrolled and 0.20 in those controlled, p = 0.10) (Supplementary Table 1).

In a sex-stratified analysis of factors associated with blood pressure control (Supplementary Table 2), the average non-adherence score remained strongly associated for men (0.05 in those with control versus 0.59 in those without control, p = 0.009). Although women with uncontrolled hypertension had higher non-adherence scores compared to women with controlled blood pressure (0.62 versus 0.32), this difference did not achieve statistical significance. Men with controlled hypertension were on ART for a median of 10.7 years versus 6.6 years for those with uncontrolled hypertension (p = 0.01); however, there was no significant difference in ART duration for women (6.2 versus 6.8 years in controlled versus uncontrolled women, respectively, p = 0.81).

### Factors associated with incident hypertension

Individuals with incident hypertension were slightly older (46 versus 42 years, p < 0.001), and had a higher body mass index (25.3 versus 22.3, p < 0.001) compared to those who did not have incident hypertension (Table 6). In a sex-stratified analysis of incident hypertension, older age and higher body mass index were significantly associated with incident hypertension for both men and women. Men with incident hypertension had a longer duration on ART compared to men who did not (6.5 versus 5.1 years, p = 0.047), while duration on ART was similar for women. Daily added salt use was high among men and women with and without incident hypertension (>90%). Sex stratified results are summarized in Supplementary Table 3.

**Table 6:** Factors associated with incident hypertension during one year of follow-up^.

	Overall n = 513	No incident hypertension over one year* n = 411	Incident hypertension over one year** n = 102	p-value

Median age (IQR)	43 (38, 50)	42 (37, 49)	46 (40, 54)	<0.001
Female sex, n (%)	242 (47.2%)	194 (47.2%)	48 (47.1%)	0.979
Median years on antiretroviral therapy (IQR)	5.6 (3.9, 7.7)	5.4 (3.8, 7.6)	6.3 (4.2, 8.0)	0.083
Highest level education completed, n (%)				
*Primary school or less*	301 (58.7%)	239 (58.2%)	62 (60.8%)	0.162
*Secondary*	148 (28.8%)	125 (30.4%)	23 (22.5%)	
*Beyond secondary*	64 (12.5%)	47 (11.4%)	17 (16.7%)	
Cigarette smoking^a^, n (%)	23 (4.5%)	22 (5.4%)	1 (1.0%)	0.056
Alcohol use^b^, n (%)	71 (13.8%)	58 (14.1%)	13 (12.7%)	0.720
Sedentary lifestyle^c^, n (%)	139 (27.1%)	110 (26.8%)	29 (28.4%)	0.735
Daily added salt to diet^d^, n (%)	507 (98.8%)	408 (99.3%)	99 (97.1%)	0.063
Mean Body Mass Index kg/m^2^ (IQR)	22.6 (20.2, 26.1)	22.3 (19.9, 25.4)	25.3 (21.8, 28.7)	<0.001
Undetectable viral load copies within 12 months of baseline visit (<1,000 copies/mL), n (%)^e^	217 (95.2%)	179 (94.2%)	38 (100%)	0.128
Diabetes^f^, n (%)	10 (1.9%)	9 (2.2%)	1 (1.0%)	0.428

^ Includes all individuals at baseline who were not on antihypertensives (i.e. not known to be hypertensive based on chart review). * Blood pressure: <140/90 mm Hg at all follow-up or a single visit with ≥140 and/or ≥90 mm Hg. ** Incident hypertension defined as blood pressure ≥140 and/or ≥90 mm Hg at ≥2 follow-up visits after baseline (and no prior known diagnosis of hypertension). ^a^ Based on self-report of current tobacco smoking, regardless of duration or number of cigarettes per day. ^b^ Alcohol use defined as any ‘yes’ response to survey question ‘Do you drink alcohol?,’ regardless of frequency or quantity. ^c^ Sedentary lifestyle defined as spending more than half of the day seated during typical days in the past month. ^d^ Based on self-report of adding salt to food on a daily basis. ^e^ Among 228 people with viral load available. ^f^ Based on self-report.

## Discussion

We found low rates of blood pressure control over one year among a population of adults with HIV who were on long-term ART and taking antihypertensive medications. While many studies have characterized the prevalence of hypertension among adults with HIV, few have prospectively followed individuals to evaluate control over time. One such study, the Uganda SEARCH study, longitudinally followed individuals with hypertension (both with and without HIV) within an integrated chronic disease clinic [14]. Hypertension was controlled at 15% of baseline visits and 46% of post-baseline follow-up visits, which were performed over three years. In a previous study from Malawi evaluating integrated HIV and hypertension care in an urban setting, 26% of individuals had controlled blood pressure after six months of follow-up, with control defined as normal blood pressure at the last visit [15]. Our study findings of ~20% of individuals with controlled hypertension over one year is slightly lower, which may result from our more stringent definition of control compared to this prior study. Taken together, these data suggest the vast majority of individuals living with HIV and on ART and antihypertensive medication are not achieving blood pressure control and are at risk for long-term morbidity and mortality.

Risk factors for poor blood pressure control have not been well-characterized in people living with HIV and hypertension in Africa. In the aforementioned SEARCH study, men (aOR 0.88, 95% CI 0.78–0.99) and individuals over age 50 years (aOR 0.83, 95% CI 0.73–0.95) were less likely to have controlled blood pressure [14]. A case-control study from Zimbabwe of individuals with HIV and hypertension found that adding salt to dishes regularly, body mass index >25 kg/m^2^, and history of an elevated blood pressure in the prior year were all independently associated with uncontrolled hypertension [19]. Our study did not find significant differences in blood pressure control by demographic, clinical, or lifestyle factors, including by age, sex, or body mass index. We found low rates of diabetes as well as alcohol and tobacco use; therefore, we could not evaluate the role of these factors on blood pressure control.

Our study is among the first to longitudinally evaluate adherence to antihypertensive medication therapy and blood pressure control among people living with HIV in Africa. A study from Ghana in people without HIV also found non-adherence to antihypertensive medication to be associated with poorly controlled blood pressure, in addition to male sex, concurrent diabetes, a higher number of antihypertensive medications, and lower glomerular filtration rate [20]. In our study, adherence level was the only factor significantly associated with blood pressure control. This contrasts with the high rates of self-reported adherence to ART and high rates of viral suppression among those with viral load performed. The majority of individuals in our cohort reported frequently missing doses of antihypertensive medications, citing barriers such as difficulty remembering to take medications daily and feeling healthy, such that antihypertensive medications were not thought to be necessary. Additionally, side effects were common, especially for women, although they were rarely severe enough to interfere with adherence. Prior qualitative work from our group with individuals on treatment for both HIV and hypertension demonstrated that while hypertension is viewed as a serious disease with life-threatening consequences, the fact that hypertension can be controlled through lifestyle changes makes medical management feel less necessary as compared to ART [21]. Consistent with data from this observational cohort, participants in our prior qualitative study had high ART adherence and low adherence to antihypertensive medications, with this discrepancy attributed, in part, to the high costs of antihypertensive medications. Similar data from the US suggest people with HIV view ART as more necessary than antihypertensive medication and subsequently are more adherent to HIV treatment [22]. Widespread education campaigns around the importance of lifelong ART and viral suppression have resulted in high rates of knowledge about HIV and high viral suppression rates in Malawi and other settings in sub-Saharan Africa [23]. Education campaigns for NCDs, which have not been undertaken in Malawi to date, combined with patient education and counseling could be an important tool to help people living with hypertension better understand the importance of treatment, achieve high levels of adherence, and improve blood pressure control.

The cost of hypertension medications emerged as a consistent barrier for individuals in our cohort. While care for HIV is provided for free in Malawi and similar settings, free medication for NCDs is limited to specific government clinics, which can be overwhelmed with patients and often have medication stock-outs or have limited medications for the management of NCDs. At Partners in Hope, while HIV care (including ART) and hypertension screening and clinical management are provided free-of-charge, medications for NCDs were only available for purchase during the period under study. While patients can travel to a government clinic to obtain free medications, the additional time (including time away from work, childcare, or other responsibilities) and cost of transport may serve as a significant barrier to obtaining refills elsewhere. Previous research by our group showed that despite the costs of medications, integrated care in which medications for hypertension are purchased at the point of care does provide cost savings for people living with HIV due to the efficiencies created by avoiding the time and opportunity costs of seeking free medications elsewhere [24]. Real world and cost-modeling data have also shown that integrated care reduces costs from the health system perspective [25,26], and additional cost modeling work will be important to help inform policy around the implementation of NCD care for people with and without HIV. As people living with HIV age and experience one or more NCDs, attention to the delivery of affordable or free integrated care will become increasingly important. Integrating HIV and NCD care holds promise as a strategy to reduce the burden of care seeking on patients, but to date, has not resulted in significantly improved rates of blood pressure control [15,27,28]. Further research on integrated models of care that include data on blood pressure control and factors associated with control will be important for scaling models that can succeed in improving clinical outcomes.

We hypothesize several mechanisms for poor blood pressure control in this population that merit further study and may be amenable to intervention. First, it is possible that poor medication quality (substandard or falsified products), which has been documented in African settings including for NCDs [29,30,31], may be contributing to low rates of control. Second, blood pressure control may be suboptimal due to a lack of highly effective medication combinations. At least one clinical trial is now enrolling that is specifically designed to assess different combination regimens on blood pressure control in African populations [32]. In our study, clinicians did attempt multiple adjustments to antihypertensive medication regimens among those who were not achieving control during the year of follow-up. While our study was not powered to look at effective drug combinations, this remains an important and understudied issue in Africa. Finally, clinicians may not have recognized the extent of non-adherence to antihypertensive medication and therefore may have missed opportunities for counseling and education.

### Limitations

Our study sample is small and the number of participants with hypertension was smaller than anticipated, which limited our power to identify factors associated with blood pressure control, as well as to perform a robust multivariate analysis. We did not collect data on the number of individuals approached who declined study participation nor the demographics of this group, and therefore cannot determine whether those enrolled differ from those who did not join the cohort. We collected data on the prevalence of diabetes by self-report and therefore likely underestimate the true prevalence of this condition in our study population. We did not have information about whether hypertension had been formally diagnosed by a provider among those with elevated blood pressure but not on antihypertensive medication at study entry, and we could not rely on self-report due to individuals’ limited knowledge about previous blood pressure measurements. It is unclear whether these individuals had been diagnosed with hypertension and given lifestyle counseling, or whether they were truly incident cases of hypertension. This may have resulted in an underestimation of hypertension prevalence and overestimation of hypertension incidence. We could not evaluate rates of blood pressure control in those with incident hypertension due to limited follow-up time on study. All adherence data was from self-report and could result in over- or under-estimation of actual pill-taking behavior. At the time under study, viral load scale up was just beginning in Malawi with the recommendation for viral load monitoring every two years; therefore, viral load data was available for only a small subset of our cohort and we thus could not explore the relationship between viral suppression and hypertension control. This is a single center experience from an urban clinic and findings may not be generalizable to other settings.

## Conclusion

Adults living with HIV and hypertension in our cohort had low rates of blood pressure control associated with self-reported non-adherence to antihypertensive medication. Given the high prevalence and incidence of hypertension in Malawi and similar countries in southern Africa, interventions to improve blood pressure control are needed to prevent associated long-term cardio- and cerebrovascular morbidity and mortality. Further research is also needed on strategies that can help individuals overcome barriers to antihypertensive medication adherence, including evaluation of models of integrated care for HIV and NCDs. Ultimately, major gains in rates of hypertension control will be needed to optimize the benefits of ART for improving life expectancy.

## Additional File

The additional file for this article can be found as follows:

10.5334/gh.1081.s1 Supplementary Figure 1. Changes in blood pressure treatment from first study visit to final visit among participants with controlled hypertension (panel A) and participants with uncontrolled hypertension (panel B).

10.5334/gh.1081.s2 Supplementary Table 1. Factors associated with uncontrolled blood pressure over one year of follow-up in which controlled blood pressure was defined as <140/90 mm Hg at all follow-up visits.

10.5334/gh.1081.s3 Supplementary Table 2. Factors associated with uncontrolled blood pressure over one year of follow-up, stratified by sex, among individuals diagnosed with hypertension and on antihypertensive medication at baseline.

10.5334/gh.1081.s4 Supplementary Table 3. Factors associated with incident hypertension, stratified by sex, during one year of follow-up.
