# Supplementary material for: High Rates of Uncontrolled Blood Pressure in Malawian Adults Living with HIV and Hypertension
Source: Glob Heart. 2021 Dec 6;16(1):81. doi: 10.5334/gh.1081 (PMC8663744; doi:10.5334/gh.1081)
Supplement: Supplementary Figure 1. — Changes in blood pressure treatment from first study visit to final visit among participants with controlled hypertension (panel A) and participants with uncontrolled hypertension (panel B). [file gh-16-1-1081-s1.pdf]

Supplementary Figure 1: Changes in blood pressure treatment from first study visit to final visit among participants with controlled hypertension (panel A) and participants with uncontrolled hypertension (panel B).

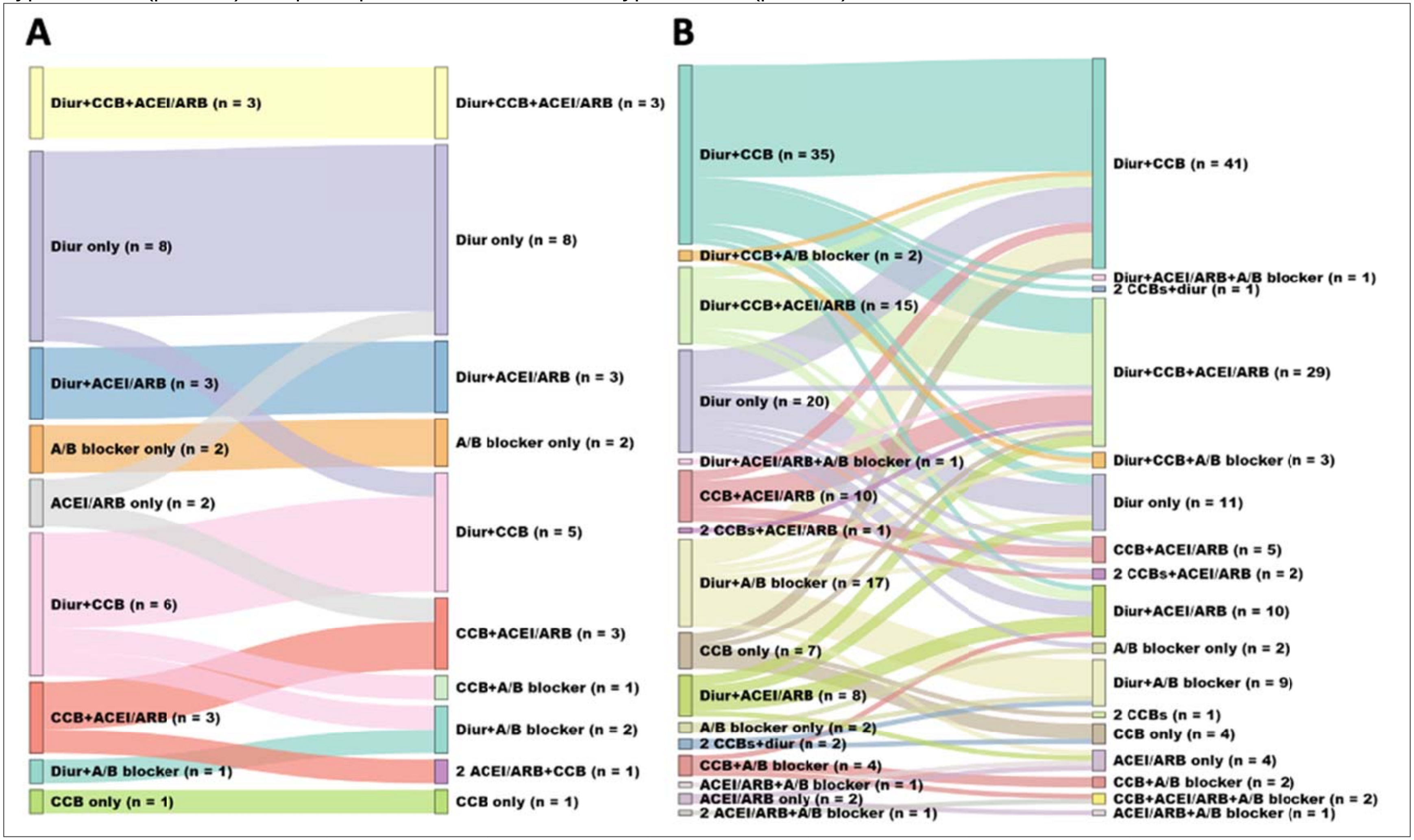

Notes: Diur=diuretic; CCB=calcium channel blocker; ACEI/ARB=angiotensin-converting enzyme inhibitor or angiotensin receptor blocker; A/B blocker= alpha or beta blocker.
