# Supplementary material for: High Rates of Uncontrolled Blood Pressure in Malawian Adults Living with HIV and Hypertension
Source: Glob Heart. 2021 Dec 6;16(1):81. doi: 10.5334/gh.1081 (PMC8663744; doi:10.5334/gh.1081)
Supplement: Supplementary Table 1. — Factors associated with uncontrolled blood pressure over one year of follow-up in which controlled blood pressure was defined as <140/90 mm Hg at all follow-up visits. [file gh-16-1-1081-s2.pdf]

Supplementary Table 1: Factors associated with uncontrolled blood pressure over one year of follow-up in which controlled blood pressure was defined as <140/90 mm Hg at all follow-up visits

	*Controlled n=12	**Uncontrolled n=146	p-value
Median age (IQR)	49.5 (42.5, 53)	51 (44, 58)	0.229
Female sex, n (%)	8 (66.7%)	97 (66.4%)	0.987
Median years on antiretroviral therapy (IQR)	8.7 (5.1, 10.0)	6.8 (4.7, 9.0)	0.315
Highest level education completed, n (%)			
Primary school or less	9 (75.0%)	75 (51.4%)	0.285
Secondary	2 (16.7%)	43 (29.5%)	
Beyond secondary	1 (8.3%)	28 (19.2%)	
Cigarette smoking[a], n (%)	0 (0.0%)	0 (0.0%)	n/a
Alcohol use[b], n (%)	0 (0.0%)	9 (6.2%)	0.376
Sedentary lifestyle[c], n (%)	1 (8.3%)	32 (21.9%)	0.266
Daily added salt to diet[d], n (%)	12 (100%)	139 (95.2%)	0.438
Average antihypertensive non-adherence score[e]	0.20	0.57	0.105
Mean Body Mass Index kg/m^2 (IQR)	23.3 (22.8, 32.1)	25.6 (21.4, 29.4)	0.942
Undetectable viral load copies within 12 months of baseline visit (<1,000 copies/mL), n (%)[f]	4 (100%)	60 (96.8%)	0.715
Diabetes[g], n (%)	2 (16.7%)	8 (5.6%)	0.131

*Controlled: normal blood pressure (<140 systolic and <90 mm Hg diastolic) at every visit
**Uncontrolled: Any visit during the one year of follow-up with a blood pressure ≥140 systolic and/or ≥90 mm Hg diastolic
[a] Based on self-report of current tobacco smoking, regardless of duration or number of cigarettes per day
[b] Alcohol use defined as any ‘yes’ response to survey question ‘Do you drink alcohol?’, regardless of frequency or quantity
[c] Sedentary lifestyle defined as spending more than half of the day seated during typical days in the past month
[d] Based on self-report of adding salt to food on a daily basis
[e] Higher score indicates higher level of non-adherence. Average weekly adherence to antihypertensive medication since last visit was self-reported at each visit and scored as follows: 0 points for missing medications less than once per week; 1 point for missing medication once per week; 2 points for missing medication two to three times per week; and 3 points for missing medication more than three times per week. Non-adherence score calculated as mean number of points per respondent across all follow-up visits.
[f] Among 66 people with recent viral load available
[g] Based on self-report
