# Supplementary material for: High Rates of Uncontrolled Blood Pressure in Malawian Adults Living with HIV and Hypertension
Source: Glob Heart. 2021 Dec 6;16(1):81. doi: 10.5334/gh.1081 (PMC8663744; doi:10.5334/gh.1081)
Supplement: Supplementary Table 2. — Factors associated with uncontrolled blood pressure over one year of follow-up, stratified by sex, among individuals diagnosed with hypertension and on antihypertensive medication at baseline. [file gh-16-1-1081-s3.pdf]

Supplementary Table 2: Factors associated with uncontrolled blood pressure over one year of follow-up, stratified by sex, among individuals diagnosed with hypertension and on antihypertensive medication at baseline

		Female			Male		
	**Overall** n=158	***Controlled** n=19	****Uncontrolled** n=86	**p-value**	**Controlled** n=11	**Uncontrolled** n=42	**p-value**
Median age (IQR)	51 (44, 57)	51 (43, 60)	50 (41, 57)	0.755	52 (44, 55)	53 (46, 59)	0.391
Median years on antiretroviral therapy (IQR)	6.9 (4.8, 9.0)	6.2 (4.6, 9.3)	6.8 (4.8, 8.4)	0.809	10.7 (6.0, 12.4)	6.6 (4.3, 9.1)	0.015
Highest level education completed, n (%)							
Primary school or less	84 (53.2%)	11 (57.9%)	51 (59.3%)		7 (63.6%)	15 (35.7%)	
Secondary	45 (28.5%)	6 (31.6%)	22 (25.6%)	0.801	2 (18.2%)	15 (35.7%)	0.243
Beyond secondary	29 (18.4%)	2 (10.5%)	13 (15.1%)		2 (18.2%)	12 (28.6%)	
Cigarette smoking[a], n (%)	0 (0.0%)	0 (0.0%)	0 (0.0%)	n/a	0 (0.0%)	0 (0.0%)	n/a
Alcohol use[b], n (%)	9 (5.7%)	0 (0.0%)	3 (3.5%)	0.409	0 (0.0%)	6 (14.3%)	0.183
Sedentary lifestyle[c], n (%)	33 (20.9%)	4 (21.1%)	19 (22.1%)	0.921	1 (9.1%)	9 (21.4%)	0.352
Daily added salt to diet[d], n (%)	151 (95.6%)	19 (100%)	82 (95.3%)	0.338	10 (90.9%)	40 (95.2%)	0.580
Average non-adherence score[e]	0.54	0.32	0.62	0.145	0.05	0.59	0.009
Mean Body Mass Index kg/m^2 (IQR)	25.5 (21.9, 29.4)	27.9 (22, 34.8)	26.2 (22.8, 31.2)	0.665	24.7 (23.1, 28.8)	23.5 (20, 26.4)	0.119
Undetectable viral load copies within 12 months of baseline visit (<1,000 copies/mL), n (%)[f]	64 (97.0%)	6 (100%)	35 (94.6%)	0.560	5 (100%)	18 (100%)	n/a
Diabetes[g], n (%)	10 (6.3%)	1 (5.6%)	7 (8.1%)	0.708	1 (9.1%)	1 (2.4%)	0.308

*Controlled: normal blood pressure (<140 systolic and <90 mm Hg diastolic) at every visit

**Uncontrolled: Any visit during the one year of follow-up with a blood pressure ≥140 systolic and/or ≥90 mm Hg diastolic

[a] Based on self-report of current tobacco smoking, regardless of duration or number of cigarettes per day

[b] Alcohol use defined as any ‘yes’ response to survey question ‘Do you drink alcohol?’, regardless of frequency or quantity

[c] Sedentary lifestyle defined as spending more than half of the day seated during typical days in the past month

[d] Based on self-report of adding salt to food on a daily basis

[e] Higher score indicates higher level of non-adherence. Average weekly adherence to antihypertensive medication since last visit was self-reported at each visit and scored as follows: 0 points for missing medications less than once per week; 1 point for missing medication once per week; 2 points for missing medication two to three times per week; and 3 points for missing medication more than three times per week. Non-adherence score calculated as mean number of points per respondent across all follow-up visits.

[f] Among 66 people with recent viral load available

[g] Based on self-report
