# Supplementary material for: High Rates of Uncontrolled Blood Pressure in Malawian Adults Living with HIV and Hypertension
Source: Glob Heart. 2021 Dec 6;16(1):81. doi: 10.5334/gh.1081 (PMC8663744; doi:10.5334/gh.1081)
Supplement: Supplementary Table 3. — Factors associated with incident hypertension, stratified by sex, during one year of follow-up. [file gh-16-1-1081-s4.pdf]

Supplementary Table 3: Factors associated with incident hypertension, stratified by sex, during one year of follow-up^

		Female			Male		
	**Overall** n=513	***No incident hypertension** n=194	****Incident hypertension** n=48	**p-value**	***No incident hypertension** n=217	****Incident hypertension** n=54	**p-value**
Median age (IQR)	43 (38, 50)	41 (35, 47)	44 (40, 52)	0.009	44 (39, 50)	48 (39, 57)	0.009
Median years on antiretroviral therapy (IQR)	5.6 (3.9, 7.7)	6.1 (4.0, 8.0)	6.3 (4.8, 7.8)	0.714	5.1 (3.5, 7.1)	6.5 (4.2, 8.4)	0.047
Highest level education completed, n (%)							
Primary school or less	301 (58.7%)	122 (62.9%)	32 (66.7%)		117 (53.9%)	30 (55.6%)	
Secondary	148 (28.8%)	47 (24.2%)	14 (29.2%)	0.214	78 (35.9%)	9 (16.7%)	0.001
Beyond secondary	64 (12.5%)	25 (12.9%)	2 (4.2%)		22 (10.1%)	15 (27.8%)	
Cigarette smoking[a], n (%)	23 (4.5%)	0 (0.0%)	0 (0.0%)	n/a	22 (10.1%)	1 (1.9%)	0.051
Alcohol use[b], n (%)	71 (13.8%)	8 (4.1%)	0 (0.0%)	0.153	50 (23.0%)	13 (24.1%)	0.872
Sedentary lifestyle[c], n (%)	139 (27.1%)	36 (18.6%)	8 (16.7%)	0.761	74 (34.1%)	21 (38.9%)	0.509
Daily added salt to diet[d], n (%)	507 (98.8%)	193 (99.5%)	48 (100%)	0.618	215 (99.1%)	51 (94.4%)	0.024
Mean Body Mass Index, kg/m^2 (IQR)	22.6 (20.2, 26.1)	23.9 (21.1, 26.9)	26.5 (24.5, 31.1)	0.0002	21 (19.4, 23.2)	23.6 (21.1, 27.5)	<0.001
Undetectable viral load copies within 12 months of baseline visit (<1,000 copies/mL), n (%)[e]	217 (95.2%)	80 (93.0%)	14 (100%)	0.308	99 (95.2%)	24 (100%)	0.273
Diabetes[f], n (%)	10 (1.9%)	3 (1.5%)	1 (2.1%)	0.794	6 (2.8%)	0 (0.0%)	0.215

^Includes all individuals at baseline who were not on antihypertensives (i.e. not known to be hypertensive based on chart review)
*Blood pressure: <140/90 mm Hg at all follow-up or a single visit with ≥ 140 and/or ≥90 mm Hg
**Incident hypertension defined as blood pressure ≥140 and/or ≥90 mm Hg at ≥2 follow-up visits after baseline (and no prior known diagnosis of hypertension)
[a] Based on self-report of current tobacco smoking, regardless of duration or number of cigarettes per day
[b] Alcohol use defined as any 'yes' response to survey question 'Do you drink alcohol?', regardless of frequency or quantity
[c] Sedentary lifestyle defined as spending more than half of the day seated during typical days in the past month
[d] Based on self-report of adding salt to food on a daily basis
[e] Among 228 people with viral load available
[f] Based on self-report
